# Risk factors for upper tract urothelial recurrence following local excision of bladder cancer

**DOI:** 10.1002/cam4.1642

**Published:** 2018-06-28

**Authors:** Ning Lin, Yu‐Peng Wu, Yun‐Zhi Lin, Xuan Tao, Shao‐Hao Chen, Zhi‐Bin Ke, Yong Wei, Qing‐Shui Zheng, Xue‐Yi Xue, Ning Xu

**Affiliations:** ^1^ Departments of Urology the First Affiliated Hospital of Fujian Medical University Fuzhou China

**Keywords:** bladder cancer, local excision, risk factor, upper tract recurrence

## Abstract

The mechanism of upper tract recurrence after local excision of bladder cancer remains unknown. This study was designed to identify risk factors for upper tract urothelial recurrence following local tumor excision of bladder cancer. To identify 76 597 bladder cancer patients, comprising 76 537 nonrecurrence and 60 recurrence patients, the Surveillance, Epidemiology, and End Results database was used. Kaplan‐Meier analysis and univariate and multivariate Cox proportional hazards regression models were used to determine the risk factors. Compared with the nonrecurrence group, the recurrence group was associated with older age, higher grade, high T stage, and higher proportional squamous cell carcinomas. Univariate Cox proportional hazards regression model showed that age, grades III and IV, T stage, and pathology were significantly associated with worse upper tract urothelial recurrence (UTUR) survival. However, after adjusting for prognostic factors, grade was no longer an independent prognostic factor in multivariate analysis. This study demonstrates that clinical prognosis of UTUR after local bladder tumor excision has significant independent risk factors that include age ≥60 years, T1 and T2 stage, and squamous cell carcinoma, and will require more careful consideration during follow‐up.

## 1 INTRODUCTION

Upper tract urothelial recurrence (UTUR) following bladder surgery is an infrequent event.^1^, ^2^, ^3^, ^4^ In patients with primary bladder cancer, the risk of UTUR is <4%.^5^, ^6^ It has been reported that the overall prevalence of transitional cell carcinoma within the upper tract urothelium following cystectomy ranged from 0.75% to 6.4%.^3^ Given the rare incidence of UTUR after bladder surgery, there are few patients from previous studies that could be examined regarding the pattern and predictive factors of upper tract recurrence and survival following bladder surgery. Thus, in our study, risk factors predicting higher rates of UTUR following bladder surgery were identified using Surveillance, Epidemiology, and End Results (SEER), a large population‐based database.

## 2 METHODS

### 2.1 Ethical standards

This study was approved by the ethics committee of the First Affiliated Hospital of Fujian Medical University. All information from the SEER database has been deidentified. Informed consent is not required for use of SEER data, as was confirmed by the ethics committee.

### 2.2 Patients

A total of 76 597 eligible patients were identified according to the following inclusion criteria: year of diagnosis from 2004 to 2014, bladder cancer as the first and only malignant cancer diagnosis, underwent local bladder tumor excision, pathologically confirmed squamous cell bladder carcinoma (not otherwise specified [ICD‐O‐3 8070/3]), keratinizing squamous cell bladder carcinoma (not otherwise specified [ICD‐O‐3 8071/3]), transitional cell carcinoma (not otherwise specified [ICD‐O‐3 8120/3]), papillary transitional cell carcinoma (ICD‐O‐3 8130/3), transitional cell carcinoma, and micropapillary carcinoma (ICD‐O‐3 8131/3). We excluded patients who were diagnosed with bladder cancer at autopsy only or at death, and those with other first primary cancers. Patients with lymph node‐positive bladder cancer and metastasis were also excluded from this study. Follow‐up times were calculated from 1 January 2004 to 31 December 2014.

### 2.3 Statistical analysis

The demographics and clinical characteristics of incorporated cases were compared between UTUR and non‐UTUR groups using the Chi‐square test. Survival curves were generated using the Kaplan‐Meier method, and the unadjusted UTUR survival rates were compared using the log‐rank test. UTUR survival was defined as the time from the date of diagnosis of bladder cancer to the date of upper tract urothelial carcinoma death. Cox proportional hazards regression models were used to calculate the adjusted hazard ratios (HRs) with 95% confidence intervals (CIs) for determining risk factors. Statistical analyses were performed utilizing R, version 3.4.1. A 2‐sided *P*‐value <.05 was considered statistically significant.

## 3 RESULTS

### 3.1 Patient demographics and clinical characteristics

A total of 76 597 patients met the eligibility criteria in this study, including 60 UTUR patients and 76 537 non‐UTUR patients. Demographics and clinical characteristics were stratified by recurrence status (Table 1). There were significant differences in the variables including age, grade, T stage, and pathology. UTUR patients were older than non‐UTUR patients at diagnosis (≥80, 51.667% vs 25.269%, respectively; *P* < .001). UTUR patients, compared with non‐UTUR patients, had higher tumor grades (grade III, 28.333% vs 15.884% and grade IV, 33.333% vs 23.569%, respectively; *P* = .006), and higher T stage (T1, 38.333% vs 27.116% and T2 31.667% vs 10.667%, respectively; *P* < .001). In terms of pathology subtype, more squamous cell carcinomas but less transitional cell carcinomas were seen in UTUR patients than in non‐UTUR patients (squamous cell carcinomas 5% vs 0.502%, and transitional cell carcinomas 95.000% vs 99.498%, respectively; *P* = .004).

**Table 1:** Characteristics of nonrecurrence and recurrence patients

Recurrence	Nonrecurrence	Recurrence	*P*‐value
Age, y			<.001
<60	15968 (20.863%)	1 (1.667%)	
≥60, <70	19859 (25.947%)	10 (16.667%)	
≥70, <80	21370 (27.921%)	18 (30.000%)	
≥80	19340 (25.269%)	31 (51.667%)	
Race			.178
Others	7218 (9.431%)	9 (15.000%)	
White	69319 (90.569%)	51 (85.000%)	
Gender			.224
Female	19036 (24.872%)	19 (31.667%)	
Male	57501 (75.128%)	41 (68.333%)	
Grade			.006
I	11868 (15.506%)	3 (5.000%)	
II	21453 (28.030%)	13 (21.667%)	
III	12157 (15.884%)	17 (28.333%)	
IV	18039 (23.569%)	20 (33.333%)	
Unknown	13020 (17.011%)	7 (11.667%)	
T stage			<.001
Ta	47619 (62.217%)	18 (30.000%)	
T1	20754 (27.116%)	23 (38.333%)	
T2	8164 (10.667%)	19 (31.667%)	
Pathology			.004
Transitional cell carcinoma	76153 (99.498%)	57 (95.000%)	
Squamous cell carcinoma	384 (0.502%)	3 (5.000%)	

### 3.2 Prognostic factors for UTUR survival (UTURS)

Univariate and multivariate Cox proportional hazards regression models were used to determine the prognostic factors for UTURS. Univariate analysis demonstrated that age, grade, T stage, and pathology were significantly associated with poor UTURS, age, T stage, and pathology, as validated in the subsequent multivariate analysis. After adjusting for prognostic factors, grade was no longer an independent prognostic factor in multivariate analysis.

### 3.3 Comparison of survival stratified by age, T stage, and pathology

**Figure 1:**
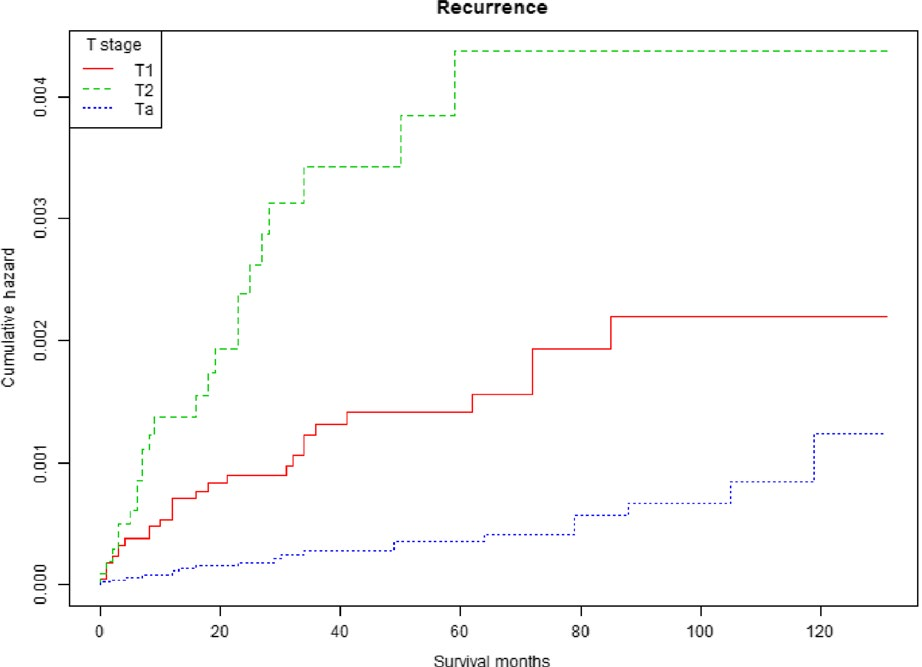
Cumulative hazard of upper tract urothelial recurrence survival stratified by T stage

**Figure 2:**
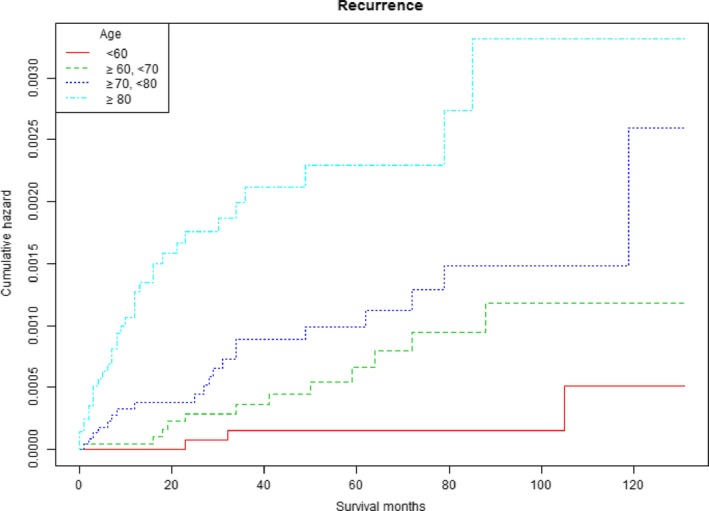
Cumulative hazard of upper tract urothelial recurrence survival stratified by age

**Figure 3:**
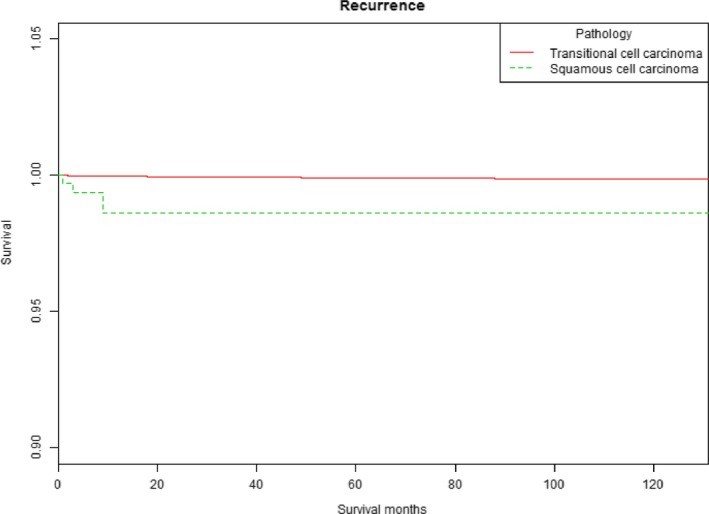
Upper tract urothelial recurrence survival stratified by pathology

Differences in UTURS among age, T stage, and pathology were estimated using Kaplan‐Meier analysis. Patients with T2 or T1 stage bladder tumors had worse UTURS than Ta stage patients (*P* < .001; Figure 1). The cumulative HR of UTUR in patients was increased with age (*P* < .001; Figure 2). The cumulative HR of UTUR in patients with squamous cell carcinomas was significantly higher than in those with transitional cell carcinomas (*P* < .001; Figure 3).

### 3.4 Stratification analysis concerning the different types of histology

**Figure 4:**
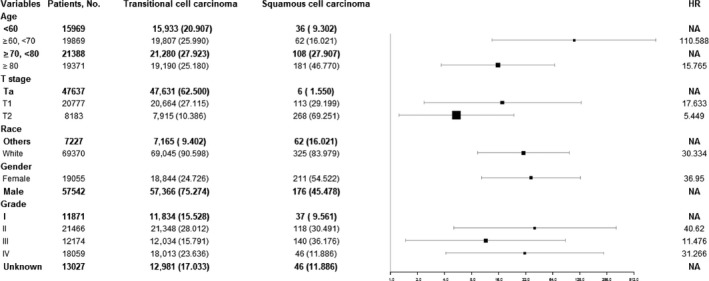
Stratification analysis of upper tract urothelial recurrence survival between transitional cell and squamous cell carcinomas

Stratification analysis of upper tract urothelial recurrence survival was performed between transitional cell and squamous cell carcinomas. Results were analyzed using a forest plot (Figure 4). As shown in Figure 4, when hazard ratios of the 2 histological groups were conducted, there were significant differences in upper tract urothelial recurrence survival, suggesting that age, T stage, race, gender, and grade may be effect modifiers of the different types of histology.

### 3.5 Comment

The recurrence rate of bladder cancer after upper tract urothelial carcinoma surgery ranges from 15% to 40%.^7^, ^8^ However, it is still controversial with respect to the recurrence rate of upper tract urothelial carcinoma after bladder surgery.^3^, ^9^

We sought to identify the risk factors for UTUR in a large cohort of patients, by utilizing SEER population‐based data. This study demonstrated that the clinicopathological characteristics of UTUR patients were different from those in non‐UTUR patients. Although risk factors for UTUR after radical cystectomy have been examined in previous studies, risk factors for UTUR after local tumor excision of bladder tumors have not been thoroughly elucidated as yet. This study revealed that age, T stage, and pathology were significant risk factors associated with UTUR after bladder surgery. However, race, gender, grade, and subtype at the time of surgery were not associated with UTUR.

In this study, T stage was a key risk factor in predicting UTUR in patients treated with local tumor excision. Cox multivariate analysis revealed that patients with T2 stage bladder cancer were at a 6.045‐fold higher risk for UTUR than those with Ta stage. Additionally, those with T1 stage were at a 2.527‐fold higher risk for UTUR than those with Ta stage. Therefore, patients with T1 or T2 stage bladder cancer who underwent local tumor excision should be monitored more closely for UTUR during follow‐up. Given that T1 and T2 stage tumors are less aggressive tumors, patients with these less aggressive tumors may live long enough to experience UTUR.^10^ Wright et al^11^ showed that low‐grade bladder cancer was predictive of a UTUR, which supports the outcome of this study.

There is currently no evidence to suggest that age plays an important role in UTUR after local bladder tumor excision. However, Cox multivariate analysis revealed that age plays an important role in UTUR after local tumor excision. Patients aged 60‐70 years showed an 8.254‐fold higher risk for UTUR than those aged <60 years. Furthermore, patients aged 70‐80 years showed a 13.494‐fold higher risk for UTUR than those aged <60 years. Finally, patients aged ≥80 years showed a 27.564‐fold higher risk for UTUR than those aged <60 years. Wright et al^11^ demonstrated that age was not predictive of a UTUR, which is in conflict with results from this study. Nathan et al^10^ performed a retrospective study of 574 patients and revealed that age was not associated with UTUR after bladder surgery, which was also at odds with this study. The HRs of UTUR were increased with increasing age, which would mean that elder people require more surveillance after local bladder tumor excision.

We also showed that patients with squamous cell carcinomas had a 6.581‐fold higher risk for UTUR than those with transitional cell carcinomas. To the best of our knowledge, this relationship has not been thoroughly studied previously. However, the different characteristics and biological behaviors between squamous cell carcinomas and transitional cell carcinomas may contribute to the reason why patients with squamous cell carcinomas had such a higher risk for UTUR than those with transitional cell carcinomas. The prognosis and natural history of patients have not yet been elucidated clearly in previous studies because of their low incidence. However, the prognosis of patients with squamous cell carcinomas was poor compared with those with transitional cell carcinomas in this study.

This study has some limitations. First, data on biomarkers were not analyzed in this study because of its retrospective nature. The relationships between the rate of UTUR and immunohistochemical and biomolecular markers should be further considered. Second, the small number of UTURs may weaken the statistical power of the analysis of risk factors. To the best of our knowledge, this is the first study to report the rate of upper tract recurrences after local bladder excision. However, it is difficult to compare the rate of upper tract recurrences in patients who underwent local bladder excision with that of patients who underwent radical cystectomy. Indeed, the rate of UTURs is low and there is a high likelihood of underreported data. Reasons that could account for the low rate of upper recurrences after local bladder excision in this study are suggested here. First, low rates of recurrence are an inherent flaw of a retrospective analysis. Second, there is no doubt that radical cystectomy offers the best opportunity for ultimate cure of high‐grade and high‐risk invasive bladder cancer.^12^ Traditionally, radical cystectomy is recommended for the majority of patients with muscle‐invasive bladder cancer (T2‐T4a, N0‐Nx, M0) with a curative intent.^13^ In addition, patients who underwent local excision of their bladder tumor in this study had a lower tumor grade than patients who underwent radical cystectomy. Thus, these patients may have lower recurrence rates than patients who underwent radical cystectomy.

In conclusion, this study demonstrates that significant independent risk factors associated with the clinical prognosis of UTUR after local bladder tumor excision include age ≥60 years, T1 and T2 stage, and squamous cell carcinoma, and will require more careful consideration during follow‐up.

## CONFLICTS OF INTEREST

The authors have declared that no competing interests exist.
